# Influence of Electric Fields and Conductivity on Pollen Tube Growth assessed via Electrical Lab-on-Chip

**DOI:** 10.1038/srep19812

**Published:** 2016-01-25

**Authors:** Carlos Agudelo, Muthukumaran Packirisamy, Anja Geitmann

**Affiliations:** 1 Optical Bio-Microsystem Lab, Mechanical Engineering Department, Concordia University, Montreal, Canada; 2 Institut de recherche en biologie végétale, Département de sciences biologiques, Université de Montréal, Montréal, Canada

## Abstract

Pollen tubes are polarly growing plant cells that are able to rapidly respond to a combination of chemical, mechanical, and electrical cues. This behavioural feature allows them to invade the flower pistil and deliver the sperm cells in highly targeted manner to receptive ovules in order to accomplish fertilization. How signals are perceived and processed in the pollen tube is still poorly understood. Evidence for electrical guidance in particular is vague and highly contradictory. To generate reproducible experimental conditions for the investigation of the effect of electric fields on pollen tube growth we developed an Electrical Lab-on-Chip (ELoC). Pollen from the species *Camellia* displayed differential sensitivity to electric fields depending on whether the entire cell or only its growing tip was exposed. The response to DC fields was dramatically higher than that to AC fields of the same strength. However, AC fields were found to restore and even promote pollen growth. Surprisingly, the pollen tube response correlated with the conductivity of the growth medium under different AC frequencies—consistent with the notion that the effect of the field on pollen tube growth may be mediated via its effect on the motion of ions.

Electric fields are known to influence a wide variety of cellular behaviour, including embryogenesis, wound healing, polarity, differentiation, and motility^1^,^2^,^3^,^4^. However, the way in which the electrical cue is perceived and translated into a cellular response is poorly understood in most cellular systems. *In vitro* experimentation has led to numerous insights, but the general lack of a systematic assessment of experimental conditions including parameters such as field type, strength and dimensions, medium composition, and conductivity, has compromised the interpretation of experimental data. To address this point and to establish a benchmark for the effects of electric fields on cell growth we have used a polarly growing cellular model system that, because of its fast growth, responds rapidly to stimuli and for which literature reports in terms of electrical behaviour have been particularly contradictory—the pollen tube.

The pollen tube is a cellular protuberance that, similar to a nerve axon or a fungal hypha, elongates by apical or tip growth (Fig. S1)^5^. It is emitted by the pollen grain, the male gametophyte in the flowering plants. It has the function to deliver the immobile sperm cells from the pollen grain attached to the stigma, through the pistillar tissues, to the ovule that contains the female gametophyte. This tip growth mechanism allows the cell to perform two important functions: mechanical invasion of a substrate^6^ and tropism (response to directional guidance cues). The target towards which the pollen tube grows can be tens of centimeters away from the location of the pollen grain that emits this cellular protuberance^7^. The precision with which the cellular elongation occurs therefore requires a complex guidance process and continuous communication between the male and female partners^8^. As growth rate is a direct selection factor for fertilization success, the pollen tube is also the fastest growing plant cell with up to 2 cm∙h^−1^ in some species. This extreme growth rate and the ability to follow guidance cues make the pollen tube an extraordinary model system for investigating cellular growth behaviour^9^. The signals that the pollen tube is believed to be able to use for navigation comprise chemical, mechanical, and electrical cues^8^. Chemical cues have been studied in detail^5^,^7^,^10^,^11^,^12^, and calcium, for example, is known to act as a chemotropic agent for pollen tubes^10^ whereas nitric oxide (NO) is a known repulsive agent^13^. Proteic signals emitted by the pistil or female gametophyte have recently been identified to guide the pollen tube *in situ*^14^. Several mechanical principles in tube growth have already been revealed^8^,^15^. The potential role of electrical stimuli for pollen tube growth, on the other hand, is poorly understood.

Plant tissues exhibit considerable electric fields^1^,^2^. DC Electric fields up to 1 V/cm have been found to occur locally, for example in the vicinity of wounds^16^, and long-distance (global) bioelectric potentials have been reported to traverse the style of pollinated plants^17^. Since these bioelectric potentials have the shape of action potentials^18^, one could argue that actual *in vivo* signals can have DC or AC components. Individual plant cells can also generate local electric fields as demonstrated in growing pollen tubes^19^. Therefore, it is reasonable to hypothesize that electric fields are involved in pollen tube growth and guidance. However, experimental findings and their interpretations have been frustratingly contradictory. Past studies have mostly relied on simply assessing a single parameter —growth direction— instead of considering the multitude of parameters that might affect the cellular response to the external electrical stimulus. In consequence, published findings are seemingly inconsistent. For instance, exposure of *in vitro* growing pollen tubes to macroscopic electric fields has shown that those of the species *Impatiens*, *Camellia japonica*, *Erythrina*, tomato, tobacco and tulip grow towards the anode^20^,^21^,^22^, whereas those of *Vinca*, *Narcissus*, *Lycoris*, *Hedychium* and *Eriobotrya* grow towards the cathode^21^,^23^,^24^. Other species such as *Gladiolus* do not exhibit any tropism^20^. Depending on the experimenter, lily pollen tubes seemed to align with the field lines of an applied electric field^25^, or not at all^20^. *Agapanthus umbelatus* pollen tubes behaved very differently depending on where in the field they were located. Under a constant electric field of 7.5 V/cm they grew towards the nearest electrode when placed in either half of the field, but oriented randomly in the center^26^.

The main reason for the seemingly contradictory findings is the variety of experimental conditions used, ranging from the geometry of the set-up, which is usually orders of magnitude larger than the analyzed cells, to the electrical parameters of the field and the chemical composition of the *in vitro* growth medium. Moreover, experimental setup descriptions and the quantification of growth parameters are often poorly detailed or justified, which seriously limits the repeatability of the tests, especially given the weak understanding of the phenomena and the great number of variables involved. Furthermore, none of the previous studies has investigated different electrical conditions in the same setup. For instance, there is no report that has systematically applied both DC and AC electric fields to the same pollen species. We therefore strived to develop an experimental setup in which the electrical parameters are precisely defined and reproducible. We anticipate that such a systematic approach to experimentation will also benefit research on other cell types displaying electro-sensitive behaviour such as crawling fibroblasts^27^,^28^, neurites^29^,^30^, roots and fungal hyphae^31^,^32^,^33^,^34^,^35^,^36^,^37^.

A critical constraint has been the difficulty in providing a testing environment that mimics key aspects of the *in vivo* growth environment of the pollen tube. The application of electric fields has always been done using a macroscopic open-assay configuration. Suitably, LoC (Lab-on-Chip) technology can provide functional devices that offer an enclosed environment that realistically resembles the pollen tube’s natural milieu in terms of microstructural conditions, which can yield more relevant results^38^,^39^,^40^,^41^. To enable multiple usage of a given chip, the device was designed to be reusable^42^. Using the ELoC (Electrical LoC) we characterized multiple features of the behaviour of *Camellia japonica* pollen tubes exposed to global and local electric fields.

## Materials and Methods

### ELoC design

**Figure 1:**
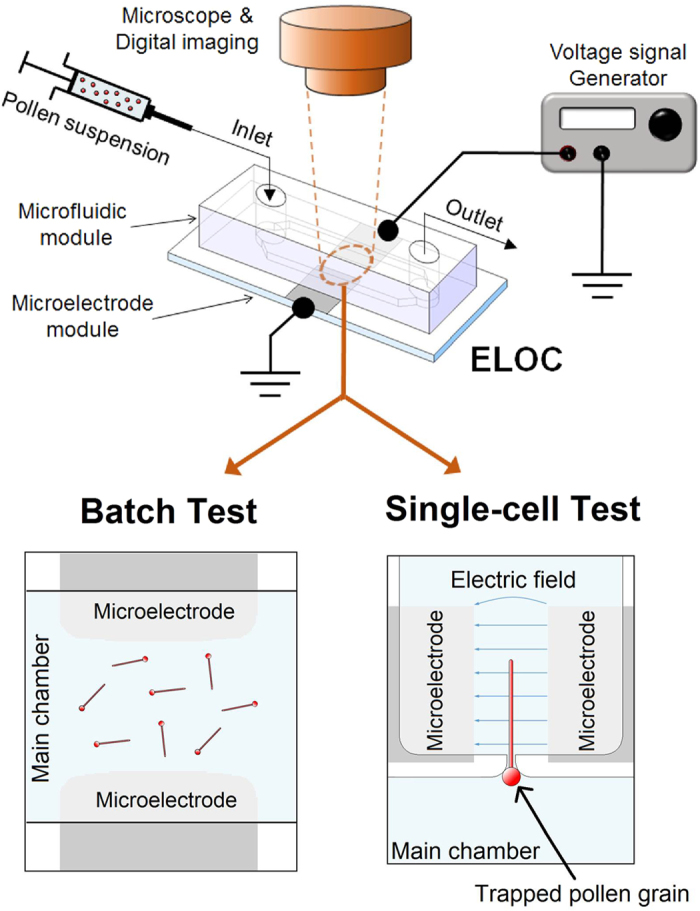
Schematic view of the ELoC set-up. The ELoC can be configured to perform both types of tests: Batch and Single-Cell.

**Figure 2:**
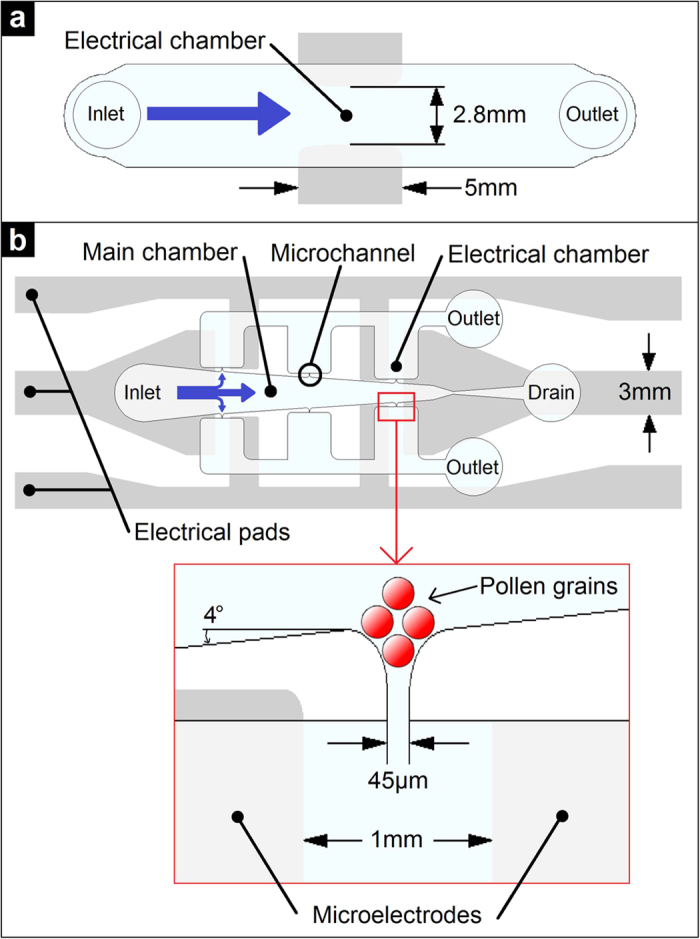
ELoC design. (**a**) Batch ELoC. (**b**) Single-cell ELoC.

The ELoC enables positioning suspended cells within microscopic electric fields and to optically monitor their growth behaviour. The integration of microelectrodes into the microdevice is technically simple and versatile, yet compact and accurate. Reusability is integrated into the ELoC by separating the design into two separate bondable modules: i) a PDMS-based microfluidic network module that accommodates the cells and the liquid medium, and ii) a microelectrode module that consists of a substrate and a metal layer through which electric fields are applied (Fig. 1). The microfluidic network layer is set to a height that is determined by the size of the pollen grains plus a margin for free movement (a total of 80 μm for *Camellia japonica* pollen). This is meant to avoid clogging while allowing free, fluid-flow driven motion of the cells. Additionally, the planar arrangement ensures compatibility with optical imaging. The geometry of the metal electrodes determines the exact electric field pattern, providing a great level of control. In order to expose pollen tubes to global and local electric fields, two different design strategies of the ELoC were considered (Fig. 2). Further design considerations and the detailed fabrication process along with a description of the reusability procedure can be found in the supplemental material.

### Biological material and experimental test conditions

*Camellia japonica* pollen was collected, dehydrated, and stored on silica gel at −20 °C until use. Pollen was thawed and rehydrated in humid atmosphere for one hour before submersion in liquid growth medium and injection into the chip. Once the pollen is positioned in the ELoC, the growth medium flow is stopped and the electric field is turned on. Then the pollen tubes are left to germinate and grow for 2 hours undisturbed unless otherwise stated. All tests were carried out at room temperature. Since the resulting electric current is small (nanoamperes in every case), heating of the medium is considered negligible.

To ensure reproducibility of test conditions, the following considerations were made: i) No flow conditions were maintained during testing. ii) The set-up eliminates the use of agarose, buffers, dyes, bridges or additives of any kind thus reducing the complexity of parameters potentially affecting the field. iii) An extreme voltage was applied at the end of every test to visually assert the presence of the electric field by inducing electrolysis. Every test series is carried out in increasing order of applied voltage to minimize any possible effect of wear in the microelectrodes due to the application of such extreme voltages. iv) Only pollen from the same plant, the same flowering season, and the same collection lot was used in a given series to minimize the effect of genetic variation, environmental conditions and developmental maturity. v) Complete wetting of each microelectrode by the growth medium is assessed visually to ensure proper electrical conduction. vi) Due to surface tension and the small LoC dimensions, it is difficult to ensure the complete absence of any trapped air, especially during injection. However, the presence of air bubbles is kept to a minimum to avoid any disruptions in the electric field distribution as a result of differences in the electrical properties of the medium. In particular, bubbles are avoided since oxygen influences pollen tube tropism^43^. Tests which did not comply with these criteria were aborted.

### Quantitative assessment of pollen tube behaviour

Pollen tube growth was monitored using an Olympus BX60M bright-field microscope equipped with a Nikon Coolpix 4500 camera (resolution of 1.28 μm per pixel). Time lapse images of the entire electrical chamber were acquired at regular intervals and stitched. The following parameters were measured: pollen tube length, percentage of germination, and percentage of bursting (tubes and ungerminated grains). As pollen tube orientation plays an important role in the putative effect of an electrical cue, tube orientation measurements under DC and AC batch electric fields were taken and can be found in the supplemental material.

## Results

### Medium conductivity

**Figure 3:**
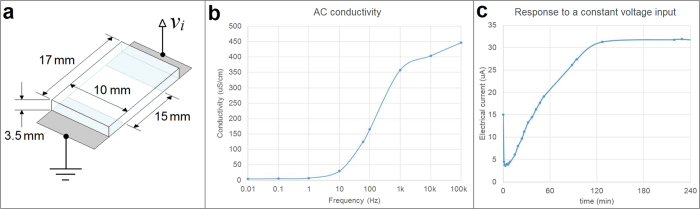
Conductivity of the growth medium. (**a**) Conductivity cell for quantification of growth medium conductivity. (**b**) Dependency of AC conductivity on frequency, (**c**) Dependency of electric current on time under constant voltage input (3.3 V).

During experimentation, the microfluidic network is completely filled with liquid growth medium, hence the behaviour of this solution in the presence of an electric field needs to be assessed. The growth medium used here was previously optimized for the species *Camellia japonica* and contains salts and sugar (1.62 mM H_3_BO_3_, 2.54 mM Ca(NO_3_)_2_·4H_2_O, 0.81 mM MgSO_4_·7H_2_O, 1 mM KNO_3_, 8% sucrose (w/v))^44^, which makes it an electrolyte. Hence, the electrical behaviour of this medium might become considerably complex when an electric field is present. Since this point is generally overlooked in the literature, here we investigated the medium conductivity in the absence of pollen to better understand the electrical behaviour of the tests. A conductivity cell was built to properly assess the bulk medium conductivity (Fig. 3a).

First, an AC conductivity test was carried out. A sinusoidal voltage input *v_i_* at different frequencies was directly applied to the conductivity cell using an Agilent 33220A waveform generator. The amplitude of the sinusoidal signal was set at 1V. For each frequency the sinusoidal electric current *i* was measured using an Agilent 34401A multimeter and the electrical resistance *R* was computed as 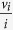. Assuming a purely ohmic behaviour, the growth medium resistivity *ρ* can be computed as 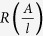, where *A* is the cross-sectional area of the conductivity cell and *l* is the length between the electrodes. Figure 3b shows the computed growth medium conductivity 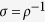 in the frequency range considered in this work. This AC conductivity test shows that the medium conductivity is highly dependent on the applied frequency. It is noted that around 1 kHz the conductivity is close to the values reported in the literature, which is consistent with the fact that most standard conductivity meters operate close to this frequency to avoid electrolysis^45^,^46^. Using standard commercial equipment (Hanna HI98129, which uses standard AC voltages) we determined a similar value for our medium (670 μS/cm). Previous experimental studies on pollen tubes reported the use of similar devices for conductivity measurement, but none of these studies actually applies an AC electric field during experimentation^20^,^22^,^26^,^47^. Figure 3b shows that as the frequency decreases, the conductivity drops. Since constant electric fields are fundamental to the present work, a DC conductivity test was carried out next.

Using the same conductivity cell, a constant voltage input (3.3V) was applied and the electric current was measured for several hours. Figure 3c shows that the electric current through the growth medium is not the constant value expected for a purely resistive medium. Initially, there is a current peak, then the current rapidly plunges, followed by a steady increase until it reaches a plateau after 2 hours. The electric current values correspond to a range of conductivities from approximately 4.6 to 41.4 μS/cm, which is considerably lower than the typical AC conductivity at 1 kHz. Two hours after the current leveled out it started to slowly decrease (down to 22 μA after 17 h). At 17 hours a scant whitish aggregation had formed along a line parallel to the microelectrodes in the center of the conductivity cell. This aggregation formed gradually and was not discernable during the first 4 hours.

### Maximum electric field conditions and simulation in DC batch tests

**Figure 4:**
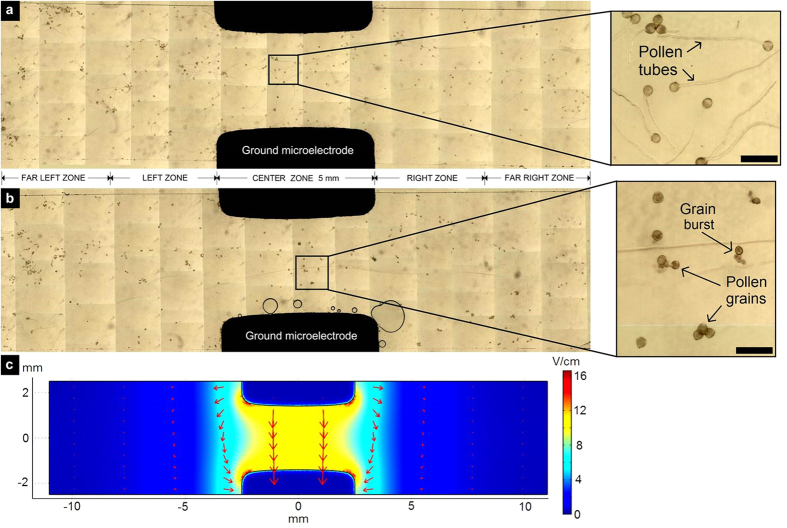
Electric field conditions and simulation. (**a,b**) Stitched micrograph of germinated pollen in DC batch electric chamber at 0 V (**a**), and 3.0 V (**b**). Scale bars on both zoom-ins = 200 μm. (**c**) Simulated electric field norm [V/cm] within the electrical chamber for a constant 3 V input. Arrows: electric field direction and normalized intensity.

In order to assess the maximum field strength that has an effect on pollen tube growth in our setup, various levels of constant voltage (up to 3.4 V) were applied and pollen tube behaviour in different zones of the batch ELoC were considered (Fig. 4). At high voltages bubbles appeared around the ground electrode (Fig. 4b). This is a by-product of electrolysis and was dose-dependent. For aluminium electrodes these bubbles started appearing after one hour of applying 3.0 V. No bubbles formed below this voltage threshold. For voltages higher than 6V, on the other hand, bubbles formed within seconds, completely covering and hence electrically insulating the microelectrode.

In order to predict the behaviour of the electrical chamber once it is filled with culture medium, 3D Finite Element Method (FEM) analysis was performed with Comsol Multiphysics. Figure 4c shows the simulated electrostatic field in the electric chamber considering the culture medium as purely ohmic with conductivity *σ*. The equation governing the physics is the Laplace’s equation:


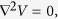


where *V* is the voltage at any particular point in the simulated geometry with respect to ground. The electric field *E* is uniquely determined by the applied voltage (as 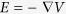) regardless of the medium conductivity. In Fig. 4c the boundary condition for the upper electrode was given as a constant 3V input value, the bottom electrode is set as ground and the limits of the geometry are set as electric insulation. Based on simulations, a slightly curved shape was given to the microelectrodes to minimize the fringing field. It is noted that the electric field between the microelectrodes (the center region of the electrical chamber) is fairly homogeneous and can be approximated by 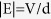, which in this case results in 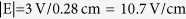. Based on this simulation, we categorized five vertically separated zones: the Center zone with a homogeneous field that is approximated by 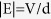; a Left and a Right zone adjacent to the parallel electrodes where the electric field fringe dominates; and a Far-Left and a Far-Right zone where the electric field is relatively small.

### Effect of batch DC electric fields on pollen tube growth

**Figure 5:**
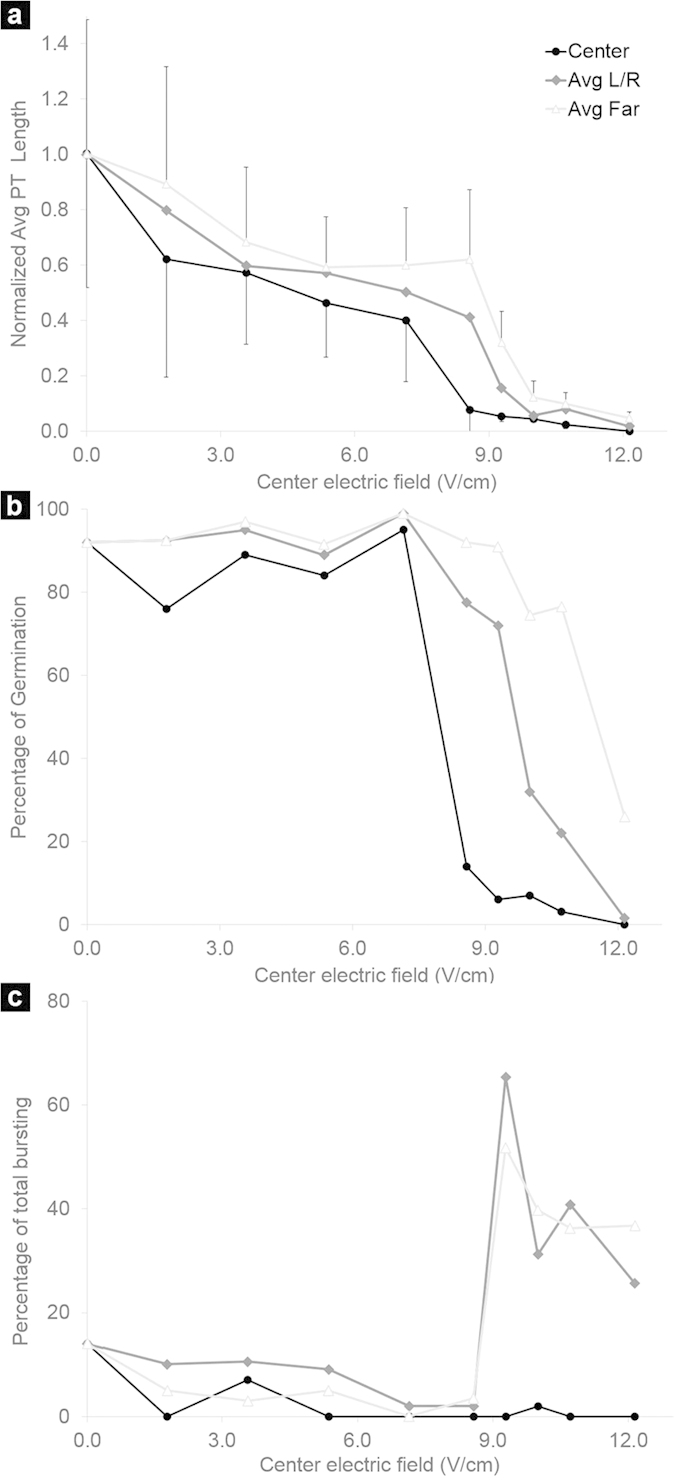
Effect of batch DC electric fields on pollen tube growth (n = 2572). (**a**) Effect on normalized pollen tube length. (**b**) Effect on percentage of pollen germination. (**c**) Effect on percentage of bursting. The field strength applied between the electrodes is indicated on the x-axis. L/R =pooled data from Left and Right Zones, Far = pooled data from Far left and right zones. For clarity, the standard deviation in (**a**) is only indicated for the Center and Far zones.

The ELoC was used to test the effect of different DC electric field strengths on pollen tube growth (Fig. 5). Each point in the x-axis corresponds to a constant electric field applied to a batch of pollen. The first point in the series corresponds to the test where no electric field is applied (hereafter called the zero-voltage test). The average pollen tube length for the zero-voltage test resulted in 721 μm, which corresponds to an average growth rate of approximately 8 μm/min (taking into account a typical germination time of 30 min). These values are well within the range reported in the literature, however they may vary for individual series (e.g. due to pollen from a different flowering season). Therefore pollen tube lengths in Fig. 5 were normalized with respect to the zero-voltage test. Pollen density in the suspension is adjusted to obtain a minimum of 25 pollen grains in each zone of the electric chamber (for statistical purposes), but no more than 80 to avoid overcrowding which would compromise imaging and might lead to cell-cell interactions. The exact number of pollen tubes analyzed per batch and per zone are provided in the supplemental material (Table S3).

Tests run at different constant electric fields showed that pollen tube performance was both dose- and position dependent. In the zero-voltage test all zones within the electric chamber showed a similar average tube length indicating that the simple vicinity to the aluminium electrode did not affect pollen tube growth (Fig. 5a). Increasing electric fields decreased the average pollen tube length. At all applied voltage levels the pollen tubes most affected were those in the Center zone, where the cells were located directly between the electrodes. The effect was less severe for the Left and Right zones and much reduced for the Far-Left and Far-Right zones. This pattern is consistent with the electric field distribution shown in Fig. 4c. When the electric field applied was sufficiently high all pollen in the LoC was effectively inhibited from growing (>12 V /cm in Fig. 5a).

Similarly to the pollen tube length, the percentage of pollen germination also decreased as the applied electric field increased (Fig. 5b). However, germination remained relatively unaffected for small electric fields but dropped steeply once the electric field was raised past a certain threshold (around 8 V/cm). Again the Central zone was more affected than the periphery. Interestingly, the pollen grains located in the Center zone did not display much bursting (always below 20%) (Fig. 5c). However, in the adjacent Left and Right zones, bursting increased dramatically for field strengths above 8 V/cm. The reason for this is elusive, but at least two considerations can be made: i) Bursting might require an ongoing germination process, whereas totally inhibited grains that do not initiate the germination process, or the accompanying softening of the cell wall in the aperture region, might not burst at all. Consequently, the range of electric field strengths that produced bursting before affecting germination in the Central zone might have been below our field strength resolution, thus reducing the total percentage of bursting events. ii) The peripheral regions of the field correspond to the nonhomogeneous portion of the field (the fringing field). The fact that bursting occurred more frequently here may indicate that this inhomogeneity has a destabilizing effect on pollen grains or on the ion gradients. In either case, this observation may warrant further investigation.

### Effect of batch DC electric fields on the instantaneous growth rate

**Figure 6:**
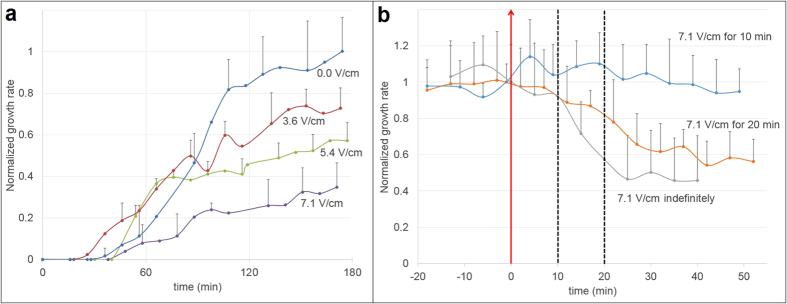
Effect of batch constant electric fields on instantaneous pollen tube growth rate. Error bars represent the standard deviation.(**a**) Normalized growth rate for increasing values of the applied constant electric field versus time (n = 13). (**b**) Normalized growth rate when a 7.1 V/cm DC electric field is applied for 10 min, 20 min, and indefinitely. Time zero (red arrow) indicates the moment at which the electric field is switched on. Dashed lines indicate the moment at which the electric field is switched off (n = 18).

To assess the effect of a batch DC field on the instantaneous growth rate, the length of individual tubes located in the center zone was monitored over time in an independent test series (Fig. 6a). The growth rates were normalized with respect to the highest average value in the zero-voltage test (10.94 μm/min in this series). Time lapse images were taken every 5 minutes from pollen injection for three hours. The data show that pollen tube growth initiated slowly and accelerated during the first two hours of the experiment, after which it reached a plateau. As the electric field increased, growth rates were reduced both during the acceleration phase and the plateau. Consistent with the previous section, an electric field above 10.7 V/cm completely prevented growth.

In order to assess the effect of the duration of field application, the test protocol was modified (Fig. 6b). Initially, pollen tubes were allowed to grow without any field applied. After 150 min, when a plateau growth rate had been reached, a strong constant field was applied (7.1 V/cm) for 10 min, 20 min, or indefinitely. The growth rate was normalized with respect to the average growth rate just prior to the electric field application: 5.59 μm/min, 6.30 μm/min, and 4.77 μm/min respectively. The data show that application of the field for 10 min did not alter the growth rate, whereas an application for 20 min caused the growth rate to decrease. The growth rate continued to decrease despite the field being switched off and a new steady value was reached (approximately 30 min after switching on the field). Over the period of observation the growth rate did not recover. When the electric field was kept on indefinitely the growth rate continued to decrease to an even lower steady value. For higher field strengths the growth rate not only decreased more dramatically, but pollen tubes tended to burst.

### Effect of batch AC electric fields on pollen tube growth

To assess the effect of a field with nonzero frequency, AC electric fields were employed. The voltage signal applied was of the form *v*(*t*) = *A·sin*(2*πft*), where *v* is the applied voltage as a function of time *t*, *A* is the sinusoidal wave amplitude, and *f* is the sinusoidal wave frequency. The same Batch ELoC and general conditions described for the DC batch tests were used. Three voltage amplitudes were selected: 1V, 2V, and 3V (which correspond to 3.6 V/cm, 7.1 V/cm, and 10.7 V/cm respectively) in order to cover the range of electric field strengths used in the DC batch tests. For each amplitude, tests were run at 0 Hz (or DC), 0.1 Hz, 1 Hz, 10 Hz, and 100 Hz. It is noted that the instantaneous electric field is still determined by the applied voltage as under DC conditions regardless of the time-varying nature of the input signal since the frequencies considered here are well within the electromagnetic quasistatic regime. The AC electric field equivalent to a given DC electric field (in the sense of the same power being delivered) is obtained by dividing the sinusoidal amplitude *A* by 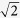.

**Figure 7:**
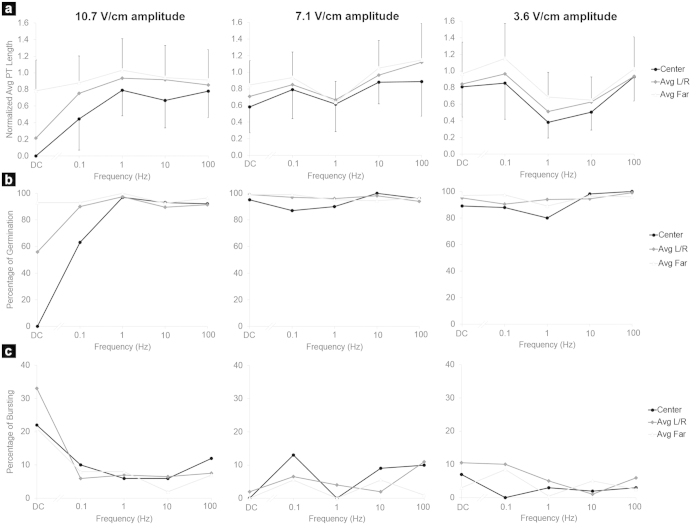
Effect of batch AC electric fields on pollen tube growth (n = 3362). (**a**) Average pollen tube length in different zones of the chamber normalized to the average control value (510 μm). (**b**) Percentage of germination. (**c**) Percentage of pollen bursting. The frequency applied is indicated on the x-axis. Each column corresponds to a different field amplitude. L/R = pooled data from Left and Right Zones, Far = pooled data from Far left and right zones. For clarity, the standard deviation in a) is only indicated for the Center and Far zones.

In the series shown in Fig. 7, the average tube length throughout the LoC for the zero-voltage test was 510 μm (Table 1). Therefore, pollen tube lengths were normalized with respect to this value. The exact number of pollen tubes analyzed per batch and per zone can be found in the supplemental material (Table S4). As expected, all tests at zero frequency resembled the DC behaviour described in the previous section. Intriguingly, the application of a nonzero frequency was increasingly beneficial for pollen tube growth. As frequency increased, the pollen tube length in all zones approached that of the zero-voltage control (Fig. 7a). This does not mean that high frequency AC fields were always without inhibitory effect since at a sufficiently high voltage (8V amplitude, which corresponds to field strength of 28.6 V/cm) the application of a 100 Hz AC field resulted in complete growth inhibition in the Center zone (Table 1). It must be emphasized that the pollen grains are still exposed to the same instantaneous electric field strengths as those of the DC fields (up to 10.7 V/cm); it is the use of a frequency that permitted the pollen to recover its normal growth. Also, Fig. 7b shows that the percentage of germination in AC fields was indistinguishable from the zero-voltage control for every amplitude and frequency tested, except for the case of a 10.7 V/cm amplitude field at very low frequencies. The same was true for the percentage of bursting (Fig. 7c). It is noted that no electrolysis was observed under any AC field.

**Figure 8:**
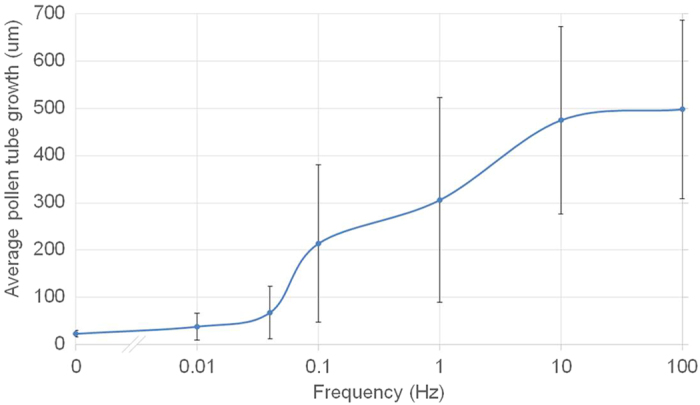
Average pollen tube length under an AC electric field in the center zone with a 10.7 V/cm amplitude (n = 245). Error bars represent the standard deviation. Frequency is plotted in logarithmic scale.

To better assess the relationship between frequency and growth behaviour we ran a new data series with field amplitude of 10.7 V/cm, a level that completely inhibited growth in the Center zone when a zero frequency (DC) is administered (Fig. 8). The tested frequencies included 10 mHz and 40 mHz, since these correspond to the inherent periodic growth oscillation in *Camellia* tubes^48^. The data show that as frequency increased, growth was promoted. Frequencies below 100 mHz had the same effect as DC, whereas growth is fully restored above 10 Hz.

### Effect of the electrode material on the pollen growth rate

The tests described above were conducted with electrodes fabricated from aluminum. In order to verify possible effects of the nature of the microelectrode material, independent tests with microelectrodes fabricated from copper and gold were carried out. A zero-voltage test under no-flow conditions in a batch ELoC built with copper micro electrodes, instead of aluminium, resulted in pollen not germinating in the Center zone. This effect has already been observed^49^. Pollen tubes grew only in the Far-zones. However, growth in the Central zone was partially restored when culture medium was continuously pumped through the inlet at 15 μl/min. Similar inhibiting effects (18% pollen germination, 29 μm average tube length in Center zone) occurred when gold electrodes were used in a zero-voltage test (no-flow). Moreover, no-flow DC tests with gold electrodes were also run with 1V and 2V yielding similar growth inhibition. Yet, when an AC electric field (1V amplitude at 100 Hz) was applied with gold electrodes (no-flow), growth was promoted dramatically (82% pollen germination, 302 μm average tube length in center zone).

### Response of pollen tubes to subcellular electric fields

**Figure 9:**
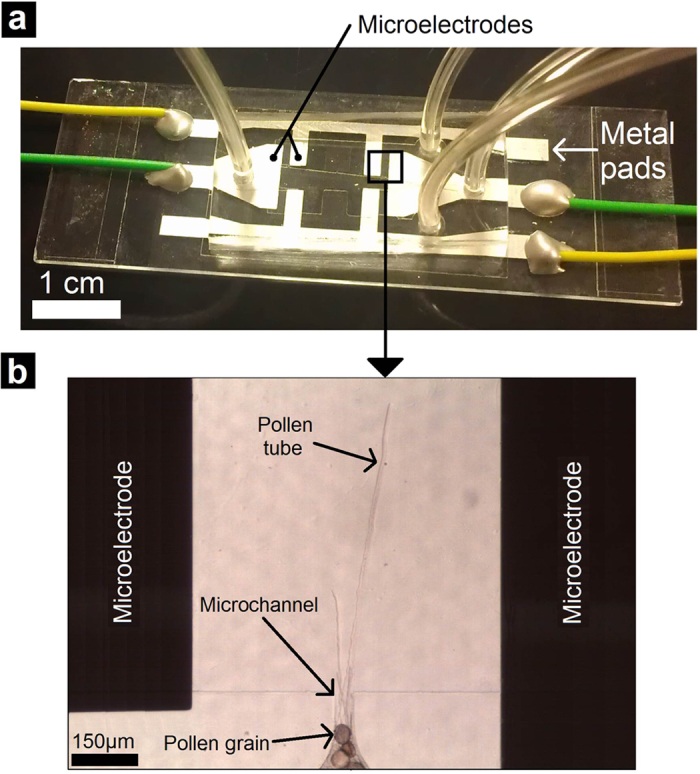
Subcellular electric field application. (**a**) Fabricated Single-cell ELoC. (**b**) Trapped pollen grains and exposure of pollen tubes to a local electric field.

**Figure 10:**
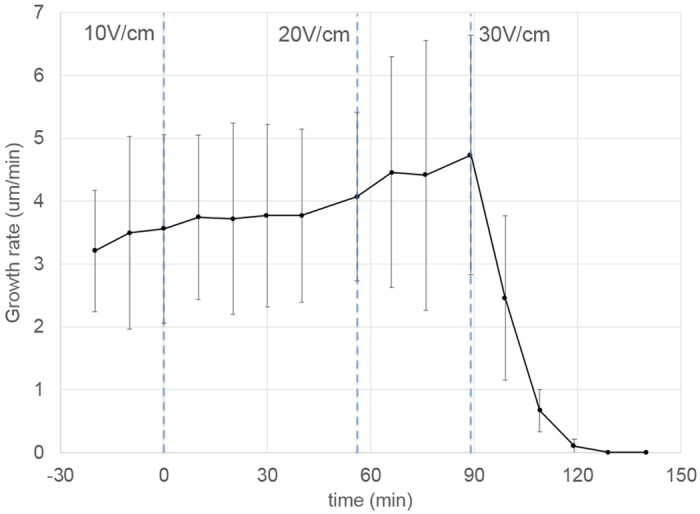
Effect of a DC electric field on the instantaneous growth rate of single pollen tubes. Time-zero indicates the instant when the initial electric field is applied. The dashed lines represent the time at which the constant electric field is increased. Error bars represent the standard deviation (n = 5).

The experiments performed on batches of pollen suggested that the processes of grain germination and pollen tube growth are subject to distinct regulatory mechanisms. We therefore designed an experimental assay in which only the growing region of the pollen tube, not the grain, would be exposed to an electric field. The single-cell ELoC was therefore used to apply electric fields in a highly localized manner, perpendicular to the growth direction (Fig. 9). In order to identify the effect on the growth rate of single pollen tubes when a DC electric field is applied only to the tip of the cell, pollen was injected into the single-cell ELoC and left to germinate with no electric field applied. Most of the pollen tubes grew into the main chamber, but a few were successfully directed to the entrance of the microchannels. By the time pollen tubes grew past the microchannels into the electric chamber (2-3 hours after injection), a steady growth rate had already been attained (indicated in Fig. 10 by the measured growth rate before time-zero).

Once the growing pollen tube tip was well between the microelectrodes, an initial 10 V/cm constant electric field was applied at time-zero during approximately 1 hour. Figure 10 shows that the growth rate was essentially unaffected by the 10 V/cm field even though the same field strength caused growth inhibition in the batch configuration. Interestingly, the growth rate remained relatively steady when the electric field was later increased to 20 V/cm. However, when a 30 V/cm electric field was applied, growth was promptly arrested. As a control, we measured the average growth rate of pollen tubes growing in the main chamber (i.e., outside of the influence of the microelectrodes). These were measured as 3.33 μm/min and 3.65 μm/min (n = 6) at t = 40 min and t = 144 min, respectively, thus indicating stable growth during this stage of pollen tube development.

### Pollen tubes do not avoid electric fields

**Figure 11:**
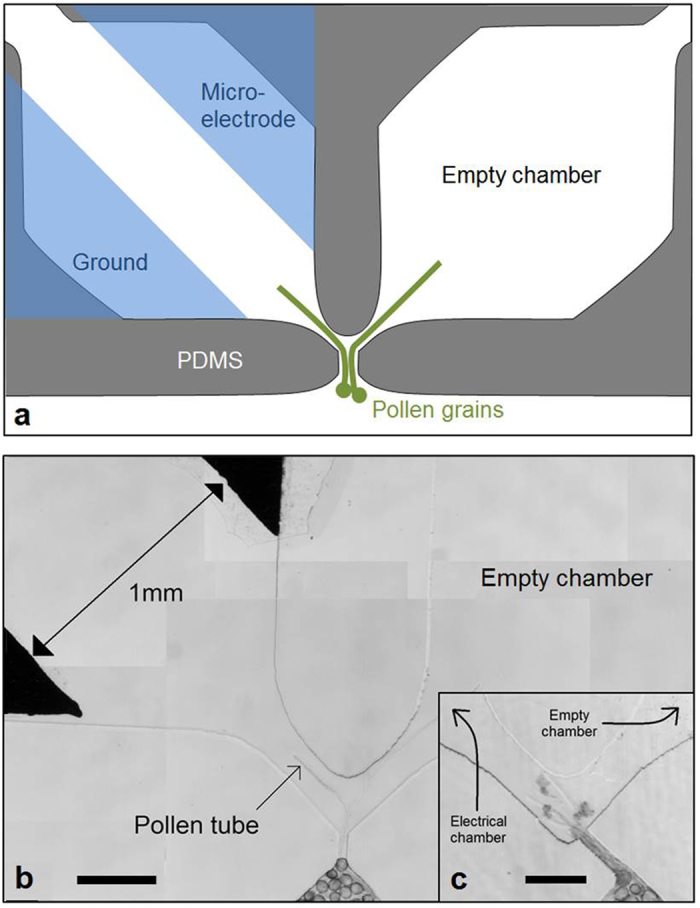
Electric field as potential guidance cue. (**a**) Design of a microfluidic network for exposure of single pollen tubes to a local, micron-scale electric field. (**b**) Micrograph showing pollen tubes growing from the microchannel into the chambers while constant voltage of 0.125 V is applied in the left chamber. (**c**) Microchannel is tilted to point directly towards the chamber containing the electric field. Scale bar = 200 μm (**b,c**).

Since the *Camellia japonica* pollen tubes located within a homogeneous electric field did not exhibit significant reorientation or alignment with respect to the electric field, we wanted to assess whether the tubes would at least sense and respond to a highly localized, micro-sized field. This test would also enable us to determine if a local electric field has the potential to act as an attraction or repulsion cue. To accomplish this, a single-cell ELoC was designed to trap and guide pollen tubes through a short, narrow microchannel (Fig. 11). If the pollen tube has any means of detecting the presence of a local high electric field as a negative directional signal, it is reasonable to expect some kind of avoidance mechanism similar to that observed in the response to certain chemical cues such as nitric oxide^13^. The microchannel was designed to point the pollen tube directly towards the local field but the microfluidic network offers the tube the option to turn away from it (Fig. 11c).

To perform the test, constant voltage between the two microelectrodes in the left chamber was applied once pollen grains were positioned within the device. The field strength was predicted to be highest in the space between the electrodes and to gradually decrease in direction of the microchannel. As the input voltage increases, the influence region of the electric field expands. Experimentation showed that at very high electric fields the effect clearly extended all the way to the microchannel since in this situation pollen tubes traversing the microchannel arrested growth. Therefore the test was conducted at moderate field strength. In the presence of an intermediate voltage (2V), which produced an electric field in the direction ahead, pollen tubes did not make any attempt to turn away from the field. Despite the increasing field strength they must experience upon approaching, they continued straight and eventually displayed the symptoms established in the batch assay such as bursting or growth arrest (Fig. 11c).

Since the presence of a local electric field did not produce any clear attraction or repulsion effect on pollen tubes when they were headed straight towards an electric field, we modified the single-cell ELoC design to offer the pollen tube a geometrically equally weighted choice by introducing a path fork with one branch leading to an electrical chamber and the other leading to an identically shaped chamber without electric field (Fig. 11a). If the presence of the electric field acts as an attractant or repulsive cue, the probability of the tube growing towards this side should be significantly different from 50%. Controls consisted in a chamber set identical to the one described but without any microelectrodes in either fork branch. In the presence of a moderate electric field in one branch of the fork, pollen tubes did not behave differently from the control (Table 2, Fig. 11b). The probability of a tube to grow towards either fork branch was indistinguishable (two-tailed t-test, P > 0.78). For 2.5 V and 3 V no pollen tube grew sufficiently long to elongate beyond the fork and they tended to burst, indicating that the electric field was already too high to allow growth inside the chambers.

## Discussion

The presence of DC electric fields on the behaviour of *Camellia* pollen tubes interfered with pollen germination and tube growth in dose-dependent manner as determined by both total length and instantaneous growth rate. Moderate fields also caused pollen grains and tubes to burst. Our systematic approach revealed a complex fact: AC fields restored pollen tube growth for frequencies greater than 100 mHz. Importantly, this recovery of growth was achieved under the same strong field strengths (up to 10.71 V/cm) that caused complete growth inhibition at lower frequencies and with DC fields. This indicates that pollen cells can tolerate strong electric fields and perform normal growth—as long as these are applied in form of high frequency AC fields. To our knowledge, this is the first direct comparison of the effect of DC and AC electric fields on the same cell type in any eukaryotic or prokaryotic cell.

The critical field strength that inhibited pollen performance when the entire cell (including grain) was exposed was approximately 10 V/cm. By contrast, a much stronger field of 30 V/cm was necessary to impede pollen tube growth when only the growing tip of the cell was exposed. This suggests that pollen tubes can endure stronger fields than grains. This finding may be explained by differences in ion transport behaviour in these two cellular regions, and is consistent with the extremely polar organization of the cell. Figures 5 and 10 also reveal a threshold-like relationship between growth and locally applied constant electric field. Growth is not degraded uniformly as the field strength increases, instead there seems to be a threshold above which growth can no longer take place. This switch-like behaviour could potentially be further investigated as a biological detection system^50^.

The performance of pollen tubes at higher AC frequencies is remarkable and warrants explanation. The comparison of Fig. 3b and Fig. 8 suggests that pollen tube performance in the electric field is related to the conductivity of the culture medium. The frequency-dependency profile of pollen tube growth matches that of medium conductivity. The similarity of the two curves is indicative of a direct correlation between conductivity and pollen tube growth. How medium conductivity influences cell growth remains to be explored. We hypothesize that this effect is related to the conditions under which the cell is able to uptake ions. Growing pollen tubes take up several types of ions from the medium, an essential prerequisite for growth^26^,^45^,^51^. When no electric field is applied, the ions in the medium surrounding the cell move solely by diffusion and possibly by convection. Ions entering the cell through ion channels transit these channels driven by diffusion. When a DC electric field is applied, however, the ions are accelerated directionally. The stronger the field, the more accelerated the ions. According to Fig. 3c, a DC electric field produces a sustained electric current (which is composed by ions in the culture medium), indicating a continuous flow of ions through the medium. Therefore, there is no ion exhaustion, which could have explained the inhibition of cell growth by lack of ions. We speculate that when the DC electric field is strong enough, the ions are too accelerated to enter ion channels even when these are open. On the other hand, when AC fields are applied, not only does the conductivity increase by orders of magnitude (Fig. 3b), which means that a considerable amount of ions becomes available for uptake; but also the ion movement is likely to be oscillatory rather than unidirectional, which presumably keeps the ions spatially confined, allowing them to enter the ion channels more easily.

Although the complex behaviour of medium conductivity with frequency has been acknowledged^52^, this fact has been neglected in virtually all previous studies on pollen tubes. If medium conductivity is reported at all, it is never mentioned at which frequency the measurement was taken^20^,^22^,^32^,^46^,^47^. Assuming conventional laboratory equipment, which uses AC fields to measure conductivity, there is an (unintended) mismatch in frequency since the actual experimentation is typically carried out in DC. Moreover, the ohmic assumption of the growth medium under DC electric fields is just a first approximation since it completely neglects the electrical double layer and its capacitive effect, let alone the complex chemical reactions occurring at the interface of any metal electrode and the electrolyte^53^. Furthermore, the distribution of ions within the medium depends on the geometry of the experimental chamber. Depending on the set-up, the cells could be exposed to different concentrations of ions at different locations in the same testing chamber. This could be particularly significant in the many centimeter-long macroscale chambers usually used in previous studies^20^,^22^,^26^,^32^,^46^,^47^. The inconsistency in terms of conductivity determination and experimental conditions might even considerably shift levels of current densities reported in studies using the vibrating probe^19^,^45^,^51^. To summarize, while it is beyond the scope of this discussion to debate the intricacies of the poorly understood electrochemistry of the situation, it is crucial to note that, according to our data, pollen growth is more likely to be influenced by the applied electric field through its effect on conductivity and ion distribution within the medium than by any direct interaction between the exogenous applied field and the endogenous electric field of the cell. Under this light, the role of medium ions deserves further investigation. Ratiometric ion imaging, for instance, could be used in conjunction with the ELoC in order to monitor and characterize ion movement under electric fields during pollen tube growth.

In this context, it is remarkable that the duration of the application of the electric field seems to be important for the degree of the cellular response, and that the effect lasts beyond the period of exposure. The application of a DC field that upon continuous exposure caused a reduction in growth rate by approximately 50% did not have any visible effect when applied for 10 minutes only. However, the growth rate dropped when the application was for 20 min or longer, and this effect was not reversible upon removal of the field. Also, if a short-lived, strong electric field does not affect the growth rate significantly (as demonstrated by the 10 min applied pulse), might it act by affecting some other physiological parameter? More importantly, is recovery of the growth rate possible under different circumstances? For instance, could a systematic inclusion of medium flow through the microfluidic network re-establish normal growth rates? Is the response time in pollen tube growth rate a purely internal cell mechanism, or could it also be a consequence of ion redistribution in the medium surrounding the cells? These questions might justify further investigation.

Concerning pollen tube orientation, no statistically significant evidence of a preferred orientation of the average pollen tube was found during the application of DC or AC electric fields in any of our tests. Nonetheless, sporadically an individual pollen tube exhibited a slight turn when the electric field was switched on, but at an angle well within the standard deviation of the difference angle measured in any zero-voltage test. Also, whenever this slight turn took place, it was always in a “single glitch” manner, that is, the tube slowed growth rate, turned slightly and continued growth; no accumulated turning over time resulting in a curved shape was observed in our work.

The lack of tropic response in our data contrasts with previous works on electrotropism. However, given that electrotropism studies have a high incidence of contradictory findings, this is not surprising. Various pollen species have been reported to turn in different directions: towards the cathode^20^, towards the anode^22^, towards the closer electrode^26^, parallel to the electric field^25^, no alignment at the centre of the field^26^. Other organisms exhibit a whole range of differing responses. For instance, maize roots have been reported to grow towards the anode and towards the cathode^33^,^34^. *Funaria* moss spores tend to form rhizoids towards the positive electrode, but when DC electric fields are applied across already developed rhizoids, their subsequent growth is directed towards the negative electrode^35^. Out of sixteen, eleven batches of fucoid eggs of the brown algae *Pelvetia* responded by initiating rhizoids towards the positive electrode, two batches responded by growing towards the negative electrode, and three had responses of a mixed sort^36^. *Neurospora crassa* and *Achlya bisexualis* mycelial fungi grew and formed branches towards the anode whereas *Aspergillus nidulans* and *Mucor mucedo* exhibited tropisms towards the cathode^31^. Some animal cells migrate to the cathode while their axis is aligned perpendicularly to the electric field^37^. Plausible explanations for the discrepancies in the electrically induced orientation, even in the same species, are difficult to assess mainly because the exact electrical, chemical, and mechanical conditions in which the tests are carried out are often not clearly described to allow for reproduction by a different lab. Notably, the physical nature of the medium surrounding the cells might play an important role. While an electric field is likely to induce a certain motion of ions in a liquid medium, a medium stiffened by agarose or other gelling agents may behave differently as it prevents the generation of convection. This work aims to improve reproducibility by providing a consistent elaboration on the design, the electrical conditions, and the enclosed environment offered by the LoC. Furthermore, the techniques used in this work can be generalized to any other growing cell type and allow for the inclusion of specific features in a systematic manner.

Since the toxicity of cells due to potential electrode by-products has been a concern in other studies^3^,^34^, the material of the microelectrodes deserves consideration. To begin with, it must be noted that even though the use of agarose salt bridges in macroscale setups might indeed delay any toxicity effect^20^,^22^,^26^,^31^,^35^,^36^,^54^,^55^,^56^,^57^,^58^,^59^, this technique does not prevent it. As long as there is a path for ions, the effect of any soluble material detaching from the electrodes remains active. In a microfluidic device the distance between cells and microelectrodes is significantly reduced compared to macroscopic set-ups. The viability of aluminium as a material for microelectrodes (at least for *Camellia japonica*) was advised previously^49^ and confirmed in this work with DC and AC zero-voltage tests, which showed that pollen growth in the vicinity of the material was statistically indistinguishable from that located far from the electrodes. However, this is not the case for copper, nor, surprisingly, for gold. Copper caused an inhibiting effect on pollen tube growth that could be restored by continuous medium flow injection. Even though gold has a low reactivity, it produced growth inhibition at zero, 1 V, and 2 V. Interestingly, growth was restored when an AC electric field (1 V amplitude at 100 Hz) was applied. The reason for this growth promotion deserves further systematic investigation, but at least these preliminary results suggest that any toxicity effect is not simply produced by the direct contact of the electrodes with the medium since the use of an AC signal alone can promote growth where a DC signal (or no signal at all) inhibits it.

As for the significance of electrical cues in the remarkable path finding ability of the pollen tube within the flower pistil, the single-cell tests show that pollen tubes are not attracted nor repulsed by localized electric fields. Pollen tubes do not seem to make any attempt to turn away from the field, instead they just burst or arrest growth. This suggests that electrical cues might not play a significant role in coarse tracking after all, or at least that it is negligible when compared to chemical and possibly mechanical cues. Of course, electric fields do affect pollen tube growth, and in all likelihood the effect can be further investigated by, for instance, applying electric fields to different portions of the pollen tube, something LoC technology readily enables. However, the effect of an electric field as a navigating aid seems to be unlikely in pollen tubes. As long as essential ions are available for uptake, mechanical and chemical cues seem to dominate guidance. Actually, one may argue that chemistry and mechanics are, at least in part, the effects of electrical phenomena at the atomic scale.

## Additional Information

**How to cite this article**: Agudelo, C. *et al*. Influence of Electric Fields and Conductivity on Pollen Tube Growth assessed via Electrical Lab-on-Chip. *Sci. Rep.* **6**, 19812; doi: 10.1038/srep19812 (2016).

## Supplementary Material

Supplementary Information

## Figures and Tables

**Table 1:** Effect of batch AC electric fields on pollen tube growth at field amplitudes 0 V/cm and 28.6 V/cm. The standard deviation is given in parenthesis.

**Amplitude**	**Frequency**	**Measurement**	**Center**	**Avg L/R**	**Avg Far**
28.6 V/cm	100 Hz	Normalized Avg PT Length	0.0 (0.0)	0.0 (0.0)	0.1 (0.1)
		% Germination	0	0	56
		% Bursting	18	34	64
0 V/cm	0 Hz	Normalized Avg PT Length	1.0 (0.3)	0.9 (0.4)	1.0 (0.4)
		% Germination	97	98	97
		% Bursting	3	0	0

**Table 2:** Growth orientation at microchannel fork offering local field at one side.

**Applied Voltage (V)**	**Towards field**	**Away from field**
0.125	3	3
0.25	6	4
0.375	1	1
0.5	7	6
0.75	2	2
1.0	3	2
1.5	2	1
2.0	5	7
2.5	0	0
3.0	0	0
Total	29	26
Control	33	36
