# Supplementary material for: Influence of Electric Fields and Conductivity on Pollen Tube Growth assessed via Electrical Lab-on-Chip
Source: Sci Rep. 2016 Jan 25;6:19812. doi: 10.1038/srep19812 (PMC4726441; doi:10.1038/srep19812)
Supplement: Supplementary Information [file srep19812-s1.pdf]

SUPPLEMENTAL MATERIAL

Influence of Electric Fields and Conductivity on Pollen Tube Growth assessed via Electrical Lab-on-Chip

Carlos Agudelo[1], Muthukumaran Packirisamy[1], Anja Geitmann[2*]

[1] Optical Bio-Microsystem Lab, Mechanical Engineering Department, Concordia University, Montreal, Canada. E-mail: pmuthu@alcor.concordia.ca.
[2] Institut de recherche en biologie végétale, Département de sciences biologiques, Université de Montréal, Montréal, Canada. E-mail: anja.geitmann@umontreal.ca

*Current address: Department of Plant Science, Faculty of Agricultural and Environmental Sciences, McGill University, 21111 Lakeshore, Ste-Anne-de-Bellevue, Québec H9X 3V9, Canada

ELoC Design, Fabrication and Reusability

The ELoC is designed as two separate bondable modules: i) a PDMS-based microfluidic network module that accommodates the suspended cells, and ii) a microelectrode module that consists of a substrate and a metal layer through which electric fields are applied. The microdevice offers a simple way to introduce and position the cells, to allow accurate exposure to electric fields during growth, and afterwards to remove the cells as well as any traces of growth medium. This last feature is particularly challenging since pollen is notoriously difficult to remove or dissolve (because of its extreme resilience to chemical reactions, pollen is often the only plant remnant in fossil records[1,2]), particularly in tightly enclosed environments where the tubes might easily get entangled.

The ELoC is designed to be opened, cleaned, and closed again for reuse by enabling the detachment and reattachment of the two modules without damaging or destroying the inner features. To ensure reusability, a SU-8 layer is patterned on top of the metal layer as to expose only the microelectrodes and to function as a protective top layer. This arrangement ensures that the metal layer never touches the microfluidic module, warranting a longer life to the microdevice.

Each module of the ELoC is fabricated separately. In general this approach offers the following practical advantages: i) since each module is independent, the fabrication of the ELoC is decoupled, maximizing fabrication yield and minimizing costs in case of fabrication defects; ii) the design of one module can be used along with different designs of the other module, increasing versatility without the need for further fabrication.

The microfluidic network consists of an inlet, a main chamber, microchannels, electrical test chambers, outlets and a drain. Fluid flow in the main chamber distributes the grains towards the entrances of the microchannels within which pollen tube growth is guided to the electrical test chambers. Each microchannel is 45 μm wide in order to trap pollen grains at its entrance while allowing passage to the pollen tube only. A drain is placed at the far end of the main chamber to evacuate excess pollen grains preventing their accumulation at the entrances of the microchannels. Additionally, any mechanical sensor or relevant structure could be included in the microchannels as required by the test. For instance, barriers, obstacle paths,[8] additional chambers, or even microcantilevers can be employed. Finally, outlets are placed at the end of the microfluidic network to provide an exit for the medium flow. Since the positioning of the pollen grains at the entrances of the traps is achieved by fluid flow, the geometry of the main chamber is crucial. To achieve uniform distribution of pollen grains over the serially arranged traps, here we propose a trapezoid-shaped main

chamber. By widening the inlet of the main chamber the inlet streamlines are better spread between the traps, improving cell distribution, as opposed to using a rectangular configuration.[3]

To assess the performance of different angles of the trapezoid shape of the main chamber, we experimentally evaluated the efficacy of cell trapping at the traps with 4°, and 8° inclinations of the main chamber shape and compared them to a rectangular (linear) shape (0°). As shown previously, a rectangular main chamber resulted in greater grain accumulation at the traps located near the outlet.[3,4] An 8° inclination reversed the trend by yielding a greater grain accumulation in the traps located near the inlet of the chamber. By choosing an intermediate 4° inclination, however, the distribution of pollen grains among the trap entrances was rendered nearly even. It is worth noting that the distribution pattern also depends on the geometry of the inlet and on the shapes of the traps and therefore needs to be optimized if the overall chamber geometry is altered.

The shape of the traps was designed to capture several pollen grains rather than single grains (Fig. 2b). Our preliminary tests have shown that when only a single *Camellia* pollen grain was trapped at the entrance of a microchannel, germination seldom occurred; whereas when a group of grains were clustered at the entrance, most of them produced healthy tubes. This improved performance by clustered cells has been observed before in other pollen species,[5] and even in bacteria,[6] and seems to apply to the species of pollen used here.

The metal layer of the ELoC consists of microelectrodes, electrical routing, and electrical pads. A variety of metals can be used for the microfabrication of the microelectrodes, each of which is associated with constraints, such as potential toxicity for the living cells.[7] For instance, copper was found to be toxic for *Camellia japonica* pollen tubes, whereas aluminium is not.[8] Other metals such as gold or platinum are often used due to their reduced reactivity,[9] but availability, cost, and ease of use are concerns.

In preliminary tests, deposited layers of metal were considered to build the microelectrodes. These included materials such as gold, indium tin oxide (ITO), and aluminium. Although deposited layers of these materials performed well electrically, they pose a problem for reusability. Because of the small thickness of evaporated or sputtered material (in the order of a few nanometers), the metal layer is scraped and torn apart very easily, making handling, detaching and reattaching of the modules unfeasible in the long term**.** To find a more viable solution (both in economic and practical terms), thicker layers were considered. As the thickness increases, the mechanical resistance to wear improves. Also, deposited metal layers entail high resistance paths between the microelectrodes and pads (>100Ω), whereas resistances of less than 1Ω are common with thicker layers. However, the metal layer cannot be too thick, since this would compromise the planarity of the protective SU-8 layer, which would in turn reduce the bonding between the microfluidic module and the electrical module. As for the metal material, even though gold has a slightly higher electrical conductivity, aluminium was chosen as the metal element since it is considerably more cost-effective for thick layers, compatible with microfabrication, and bio-compatible with pollen tubes. To ascertain that the layer is sturdy enough, a 15 µm aluminium thickness is used. Glass is chosen as the substrate onto which the metal layer is placed since transparency is required for microscopic visualization.

The following protocol was optimized for reliable fabrication of the ELoC with an integrated patterned aluminium thin film (Fig. S2).

- The fabrication of the microfluidic platform, is done using principles of soft lithography.[10] The exact details of the technique are described elsewhere.[11] Briefly, the microfluidic layout is used as a mask to reproduce the design on a Silicon/SU-8 mold through photolithography. Multiple PDMS replicas are then cast from the mold, detached, and diced (Fig. S2-1). Next, the microfluidic inlet and outlet are punched in order to allow the insertion of PVC tubes from the top of the chip (Fig. S2-2).
- A thin PDMS layer is coated on the glass substrate. 1 ml of PDMS (10:1 polymer base/curing agent ratio) is poured on a clean glass slide and spin-coated at 2000 rpm for 20 s (Fig. S2-3).
- The aluminium thin film is placed gently on top of the substrate. A larger glass is placed on top of the aluminium as to produce a flat layer. Then the entire assembly is clamped. Trapped air bubbles are evacuated by slight directed pressure and vacuum if necessary (Fig. S2-4).
- The clamped set is placed in the oven at 65°C for the PDMS layer to cure (at least 1h). Once fully cured, the set is unclamped and excess aluminium around the glass slide is cut away.
- In order to improve the adhesion and homogeneity between the photoresist and the aluminium thin film, plasma treatment is applied to the aluminium layer for 25 s. Immediately afterwards, positive photoresist (AZ1518) is spin-coated on the aluminium layer at 800 rpm for 20 s. The set is let to settle for 2 min to improve homogeneity, then

prebaked at 100°C for 2 min, and finally left to settle for 2 min at room temperature to allow hardening of the photoresist (Fig. S2-5).

- The photoresist is then patterned with a positive mask of the electrode configuration by standard UV exposure (Fig. S2-6).
- The photoresist is post-baked at 100°C for 2 min so as to fully crosslink, and let to settle for 2 min at room temperature for hardening. Then the photoresist is developed for 3 min. The photoresist is next annealed at 100°C for 2 min to remove residual stresses after development, and let to settle for 2 min at room temperature (Fig. S2-7).
- The aluminium layer is next wet-etched at 70ºC (on a hotplate) for 1 h. The aluminium etchant is a combination of phosphoric acid (80%), acetic acid (5%), nitric acid (5%), and deionized water (10%), which results in a smooth etched surface. Next the photoresist is easily removed with acetone (Fig. S2-8).
- Once the aluminium layer is patterned, a layer of SU-8 2035 is coated on top. The SU-8 is spun at 3000 rpm to produce a 40 μm thick protective/insulating layer. Then it is aligned and patterned as to expose only electrical pads and microelectrodes (Fig. S2-9).
- Electrical wires are bonded to the metal pads with conductive epoxy to provide electrical access to the electrical chamber (Fig. S2-10).
- The two modules are aligned and bonded together using oxygen plasma in order to seal the electrical chamber. In our setup (Harrick Plasma PDC-001), activation times between 30 s to 1 min produced satisfactory results. In order to ensure a proper bond and to avoid any leakage, the two modules are clamped together immediately after contact (Fig. S2-11).

Fig. S3a shows a fabricated Batch ELoC. Once the ELoC has been used for an experimental assay, it undergoes a simple procedure to make it ready for reuse. This procedure should be performed as soon as the test ends as to minimize contamination of the chip. First, the modules are gently detached by peeling the PDMS bulk from the microelectrode module (Fig. S3b). Each module can then be thoroughly cleaned and any residue removed. Finally, both modules are rebonded using the procedure described above. The proposed ELoC has been tested in several series of electrical tests and is generally expected to last dozens of reuses with minimum care.

Pollen Tube Growth Orientation

Quantitative assessment of pollen tube orientation

Pollen tube growth was monitored using an Olympus BX60M bright-field microscope equipped with a Nikon Coolpix 4500 camera (resolution of 1.28 μm per pixel). Time lapse images of the entire electrical chamber were acquired at regular intervals and stitched. The initial and final orientation of each pollen tube is measured with respect to the orientation of the microelectrodes. Only pollen tubes longer than twice the diameter of their grain were taken into account. Zero angle was considered to be perpendicular to the electric field (Fig. S4). Positive (counter clockwise) and negative (clockwise) angles are used, yielding the total range: $[-180°, 180°]$. Any angle computation outside this range is converted to its equivalent angle within the range. Furthermore, germination sites are scored with the value -1 when the pollen tube germinates towards the bottom microelectrode and +1 when the pollen tube germinates towards the top microelectrode.

Effect of batch DC electric fields on pollen tube orientation

To assess whether the direction of pollen tube growth is influenced by a constant field, we assessed the orientation of pollen tubes upon germination and after 2 hours in the set of batch DC electric field experiments. Only pollen tubes positioned in the central zone were considered. In Table S1 both the applied constant voltage and its corresponding electric field in the center zone, as predicted by the simulations, are specified. Since germination is insufficient at higher voltages, measurements were carried out up to 2.0 V. For each pollen tube the initial angle, final angle, and Top/Bottom measurements were quantified as explained above. The average of the angle difference in orientation, which corresponds to the final angle minus the initial angle for each pollen tube, is also indicated. Interestingly, no indication of any statistically significant preferred direction was found either upon germination or after two hours. As expected, the average initial degree for each test was close to zero. Given that the total range is from $-180°$ to $180°$, this indicates that the initial direction of the average pollen tube is essentially random. Alternatively, the zero average value could

also indicate that most tubes started their growth pointing to the zero-angle direction, but the high standard deviation clearly indicates an even distribution of the values over the available range. The average final angle for each test was also close to zero, indicating a random final orientation. Measuring the difference between the final and initial orientation of the pollen tube allowed us to estimate how much the average pollen tube changed direction while exposed to the electric field. This difference is also close to zero in average, indicating that the average pollen tube essentially maintained its direction throughout the whole test. Any changes in orientation seemed to be random. Furthermore, the standard deviation of the difference angle indicates that 68.2% of the pollen tubes deviated only within ±18.95° after germination. Consistent with this, the discrete Top/Bottom score showed an average close to zero, meaning that pollen tubes germinated randomly towards either microelectrode. To summarize, even though pollen tube length was critically affected, pollen tube orientation was virtually insensitive to the presence of the electrical field in our tests.

Effect of batch AC electric fields on pollen tube orientation

To assess whether the direction of pollen tube growth is influenced by an AC field, we also assessed the orientation of pollen tubes upon germination and after 2 hours in the set of batch AC electric field experiments. Only pollen tubes positioned in the central zone were considered (Table S2). Quantification of pollen tube orientation revealed that pollen tubes neither germinated nor grew into a preferred direction under AC electric fields.

Effect of single-cell DC electric fields on pollen tube orientation

To assess whether pollen tubes respond in tropic manner upon application of the electric field using the single-cell ELoC (Fig. 9), the orientation of the pollen tubes was measured. The angle between the growth direction and the electric field lines of each pollen tube were determined just before electric field application (initial angle), and at the end of the test (final angle). The initial angle is always close to 90° since the microchannel is directed perpendicular to the electric field lines, but a deviation from this in the final angle might indicate growth tropism. In average, the difference between the final and initial angles was measured to be 0.66° (n=12), with a standard deviation of 18.24°. These results indicate that pollen tubes essentially maintained their growth direction regardless of the presence of the electric field.

References

1. Gray, J., Chaloner, W. G. & Westoll, T. S. The microfossil record of early land plants: advances in understanding of early terrestrialization. *Philos. Trans. R. Soc. Lond. B. Biol. Sci.* **309**, 167–195 (1985).
2. Langdon, P. G., Barber, K. E. & Morriss, S. H. L.-C. Reconstructing climate and environmental change in northern England through chironomid and pollen analyses: evidence from Talkin Tarn, Cumbria. *J. Paleolimnol.* **32**, 197–213 (2004).
3. Sanati Nezhad, A. Optimization of flow assisted entrapment of pollen grains in a microfluidic platform for tip growth analysis. *Biomed. Microdevices.* **16**, 23–33 (2013).
4. Agudelo, C. G., Sanati Nezhad, A., Ghanbari, M., Packirisamy, M. & Geitmann, A. A microfluidic platform for the investigation of elongation growth in pollen tubes. *J. Micromechanics Microengineering.* **22**, DOI:10.1088/0960-1317/22/11/115009 (2012).
5. Brewbaker, J. L. & Kwack, B. H. The essential role of calcium ion in pollen germination and pollen tube growth. *American Journal of Botany.* **50**, 859–865 (1963).
6. Boedicker, J. Q., Vincent, M. E. & Ismagilov, R. F. Microfluidic confinement of single cells of bacteria in small volumes initiates high-density behavior of quorum sensing and growth and reveals its variability. *Angew. Chem. Int. Ed.* **48**, 5908–5911 (2009).
7. Li, J. & Lin, F. Microfluidic devices for studying chemotaxis and electrotaxis. *Trends Cell Biol.* **21**, 489–497 (2011).
8. Agudelo, C. G. et al. TipChip: a modular, MEMS-based platform for experimentation and phenotyping of tip-growing cells. *Plant J. Cell Mol. Biol.* **73**, 1057–1068 (2013).
9. Robinson, D. A. The electrical properties of metal microelectrodes. *Proc. IEEE.* **56**, 1065–1071 (1968).
10. Ziaie, B., Baldi, A., Lei, M., Gu, Y. & Siegel, R. A. Hard and soft micromachining for BioMEMS: review of techniques and examples of applications in microfluidics and drug delivery. *Adv. Drug Deliv. Rev.* **56**, 145–172 (2004).
11. Agudelo, C. G., Packirisamy, M. & Geitmann, A., in *Plant Cell Morphogenesis: Methods and Protocols,* Series "Methods in Molecular Biology" (eds. Žárský V, Cvrčková F) 237-248 (Springer, 2013).

Table S1. Pollen tube orientation under batch DC electric fields in the center zone. Standard deviation in parentheses.

Voltage (V)	Electric field (V/cm)	Initial (°)	Final (°)	Difference (°)	Top / Bottom
2.0	7.1	6.80 (103.33)	15.02 (101.92)	15.31 (22.10)	-0.04
1.5	5.4	2.26 (101.97)	-1.43 (109.86)	-3.70 (16.65)	-0.06
1.0	3.6	-4.50 (101.71)	-17.63 (108.21)	-13.12 (11.58)	0.00
0.5	1.8	-0.99 (97.02)	-0.07 (92.95)	0.93 (20.02)	0.03
0.0	0.0	-7.44 (99.65)	-5.11 (103.45)	2.33 (25.26)	-0.08
	Total average	-0.77 (100.74)	-1.84 (103.29)	-1.32 (18.95)	-0.03

Table S2. Pollen tube orientation under batch AC electric fields in the center zone. Standard deviation in parenthesis.

Electric field (V/cm)	Frequency (Hz)	Initial (°)	Final (°)	Difference (°)	Top / Bottom
28.6	100	Does not apply			
10.7	0	Does not apply			
	0.1	13.36 (83.51)	3.43 (92.44)	-9.93 (33.23)	0.00
	1	-10.65 (102.21)	-13.10 (97.82)	-4.43 (37.62)	0.05
	10	17.80 (112.74)	25.27 (103.61)	7.46 (35.02)	-0.07
	100	-8.30 (100.24)	-7.60 (112.07)	0.70 (32.16)	0.10
7.1	0	-5.77 (103.33)	1.19 (101.91)	6.96 (21.23)	-0.04
	0.1	-10.04 (118.63)	-15.75 (120.70)	-5.71 (30.65)	-0.13
	1	5.67 (117.56)	8.67 (114.31)	3.00 (30.32)	0.00
	10	-2.15 (91.64)	-13.31 (76.10)	-11.15 (32.39)	0.08
	100	1.58 (102.76)	-0.47 (104.15)	-2.05 (20.37)	0.01
3.6	0	-4.50 (101.71)	-17.62 (108.21)	-13.12 (11.58)	0.00
	0.1	8.18 (99.08)	7.95 (108.18)	-0.23 (31.77)	-0.09
	1	0.57 (111.15)	-1.57 (117.06)	-2.14 (47.94)	0.14
	10	-4.55 (97.30)	3.18 (99.03)	7.73 (16.72)	0.09
	100	-6.07 (108.68)	-3.53 (110.54)	2.53 (36.58)	0.27
0	0	-3.57 (106.86)	5.86 (103.82)	9.43 (27.70)	-0.07
	Total average	-0.56 (103.83)	-1.16 (104.66)	-0.73 (29.69)	0.02

Table S3. Number of pollen tubes analyzed per batch and per zone in the DC electric field series.

Electric field (V/cm)	Far Left	Left	Center	Right	Far Right	Total
12.1	55	45	40	46	42	228
10.7	42	45	55	51	45	238
10.0	34	46	57	80	41	258
9.3	17	48	45	54	54	218
8.6	55	42	63	44	62	266
7.1	72	70	53	69	58	322
5.4	52	35	31	33	26	177
3.6	31	36	37	48	40	192
1.8	67	60	43	73	42	285
0	80	76	76	78	78	388
						2572

Table S4. Amount of pollen tubes analyzed per batch and per zone in the AC electric field series.

Electric field (V/cm)	Frequency (Hz)	Far Left	Left	Center	Right	Far Right	Total
10.71	0	44	44	35	46	49	218
	0.1	42	47	29	40	34	192
	1	57	37	30	61	44	229
	10	42	30	28	49	35	184
	100	73	40	45	57	59	274
7.14	0	54	33	32	41	40	200
	0.1	53	33	25	31	38	180
	1	28	32	29	29	28	146
	10	38	43	32	54	44	211
	100	47	60	47	52	38	244
3.57	0	31	34	26	48	30	169
	0.1	28	38	25	47	38	176
	1	65	34	28	47	34	208
	10	55	42	44	51	48	240
	100	83	61	38	59	30	271
0	0	35	32	53	57	43	220
							3362

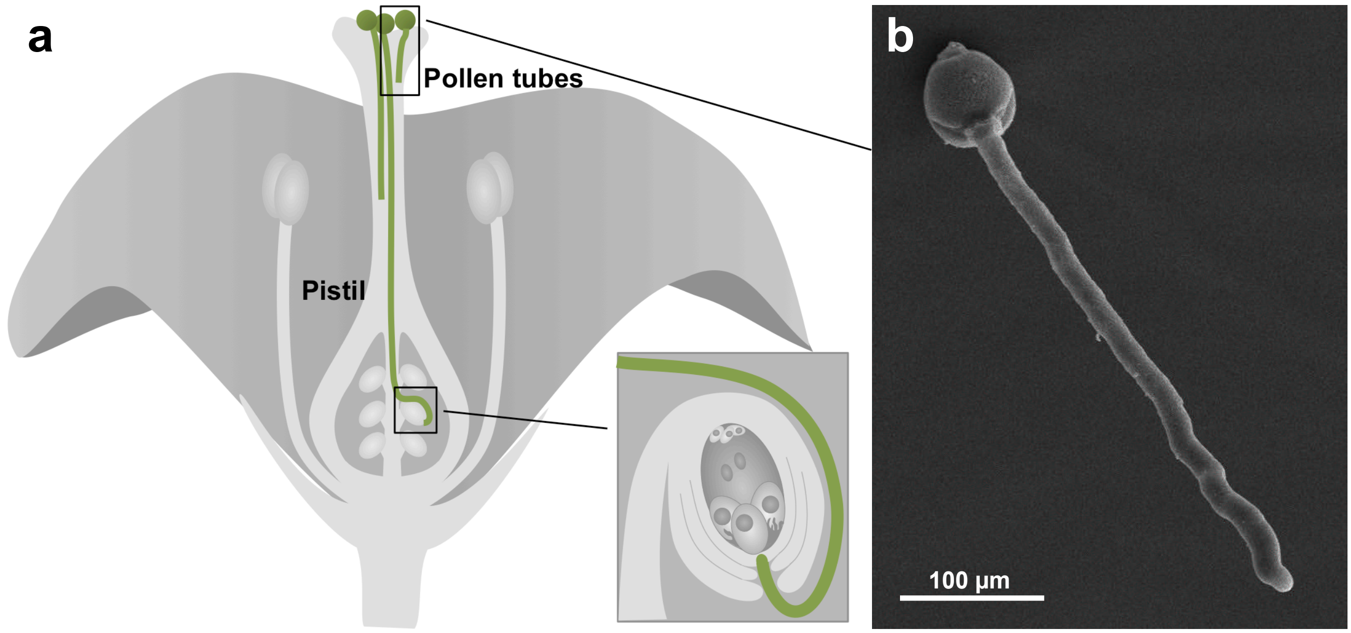

Figure S1. Sexual reproduction in plants a) Schematic view of a pollen tube growing within the pistil to reach an ovule. b) Scanning electron micrograph of a pollen tube.

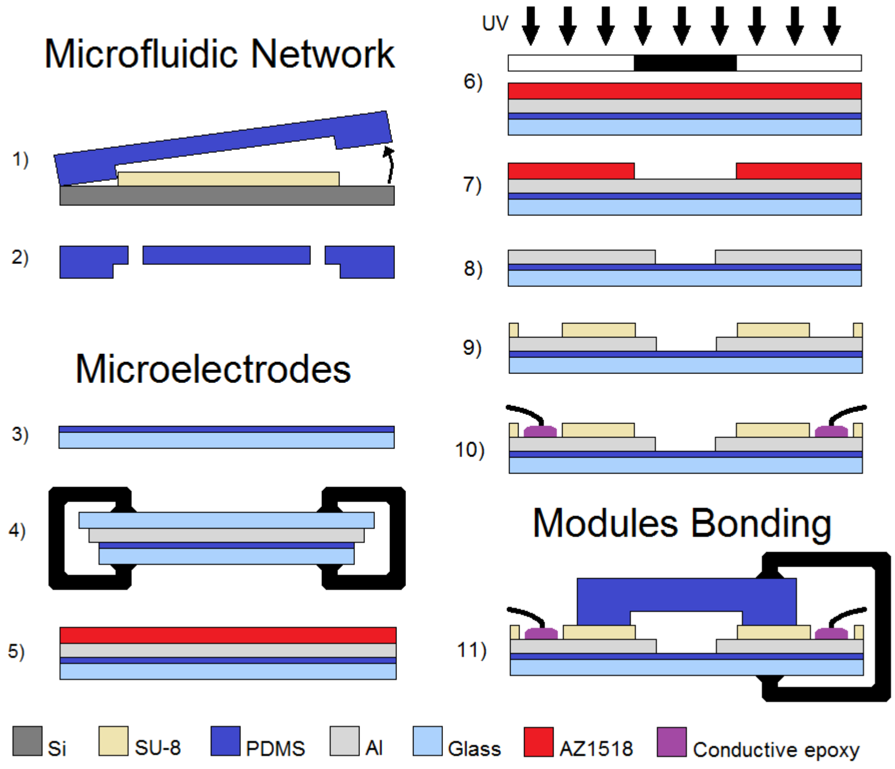

Figure S2. ELoC fabrication process: (1) PDMS layer detachment from the silicon/ SU-8 mold, (2) microfluidic inlet and outlet punching, (3) thin PDMS layer spin-coating on glass substrate, (4) aluminium thin film clamping, (5) positive photoresist (AZ1518) is spin-coated on the aluminium layer, (6) standard UV exposure with metal layer mask, (7) photoresist developing and annealing, (8) aluminium etching, (9) coating and patterning of the insulating SU-8 layer, (10) wire bonding, (11) bonding of the microfluidic network module to the microelectrode module.

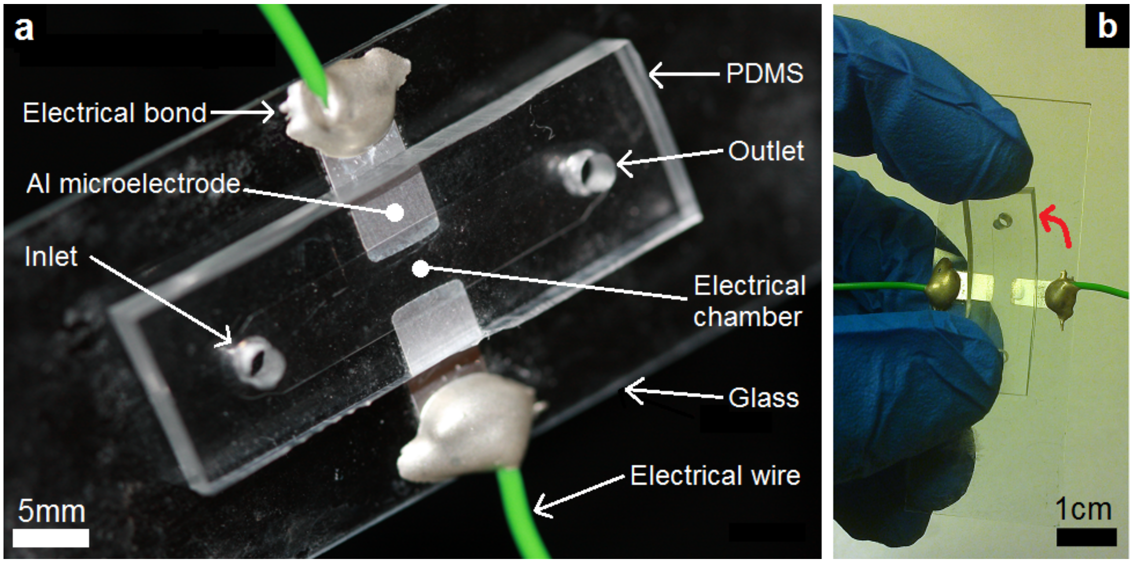

Figure S3. Reusability procedure. a) Fabricated ELoC. b) Module detachment (arrow) for cleaning and subsequent rebonding.

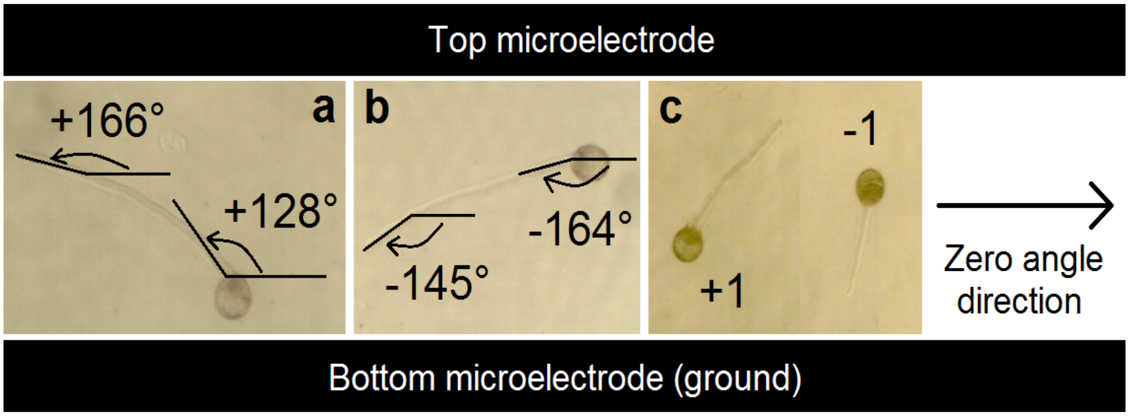

Figure S4. Quantification of pollen tube orientation. Angles were determined for the initial and final tube portions with respect to the zero angle direction as indicated by the arrow as positive angles (a), negative angles (b), and the germination site on the grain was scored with respect to its orientation towards either electrode (c).
